# Development and application of haploid embryonic stem cells

**DOI:** 10.1186/s13287-024-03727-y

**Published:** 2024-04-23

**Authors:** Hai-Song Wang, Xin-Rui Ma, Yi-Hong Guo

**Affiliations:** Center for Reproductive Medicine, The First Affiliated Hospital of Zhengzhou University, Zhengzhou University, No. 40 Daxue Road, 450052 Zhengzhou, Henan Province China

**Keywords:** Haploid embryonic stem cells, Genetic screening, X chromosome inactivation, Haploid diploidization, Gamete substitution

## Abstract

Haploid cells are a kind of cells with only one set of chromosomes. Compared with traditional diploid cells, haploid cells have unique advantages in gene screening and drug-targeted therapy, due to their phenotype being equal to the genotype. Embryonic stem cells are a kind of cells with strong differentiation potential that can differentiate into various types of cells under specific conditions in vitro. Therefore, haploid embryonic stem cells have the characteristics of both haploid cells and embryonic stem cells, which makes them have significant advantages in many aspects, such as reproductive developmental mechanism research, genetic screening, and drug-targeted therapy. Consequently, establishing haploid embryonic stem cell lines is of great significance. This paper reviews the progress of haploid embryonic stem cell research and briefly discusses the applications of haploid embryonic stem cells.

## Introduction

**Fig. 1:**
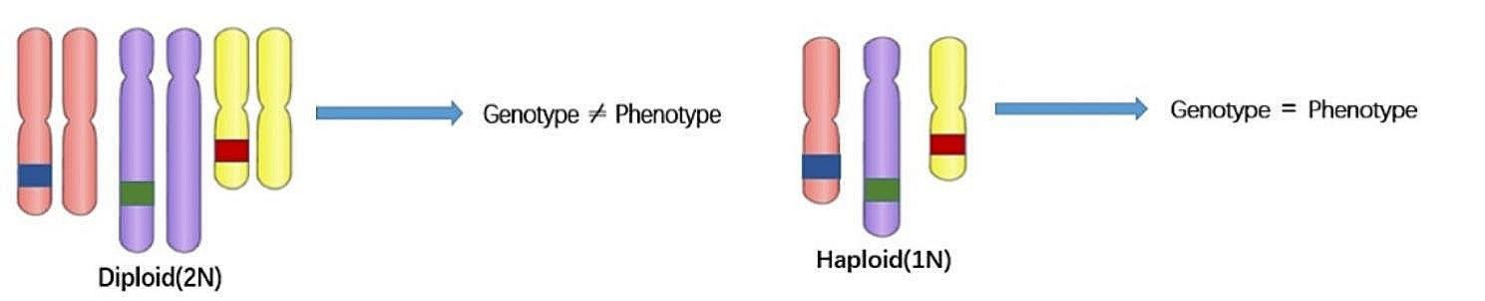
haploid compared with diploid (the blue, green, and red blots represent recessive genes)

The genetic material of most organisms in nature, including humans, contains two sets of chromosomes, one from the mother and the other from the father, called diploid organisms. Diploidy has many advantages, such as effectively preventing the elimination of organisms due to recessive deleterious mutations and maintaining the relative stability of genetics and the biosphere [1, 2]. It also increases genetic diversity through interspecific hybridization, which promotes biological evolution and the creation of new species. However, for gene research, diploidy is a great obstacle, due to the fact the phenotype of recessive genes is difficult to be reflected in heterozygotes. Therefore, for haploid cells, the characteristic that their genotypes are equal to their phenotypes gives them a unique advantage in gene research and drug screening (Fig. 1). In the natural state, only the gametes with highly specialized structures and functions are haploid cells in mammals [3, 4]. However, oocytes and sperm cannot generally be cultured and amplified for long periods in vitro, so it is difficult to manipulate them genetically. Therefore, successfully establishing mammalian haploid stem cell lines in vitro is significant in promoting mammalian genetics, development, evolution, and other related life science research.

## HaESCs acquisition history

**Fig. 2:**
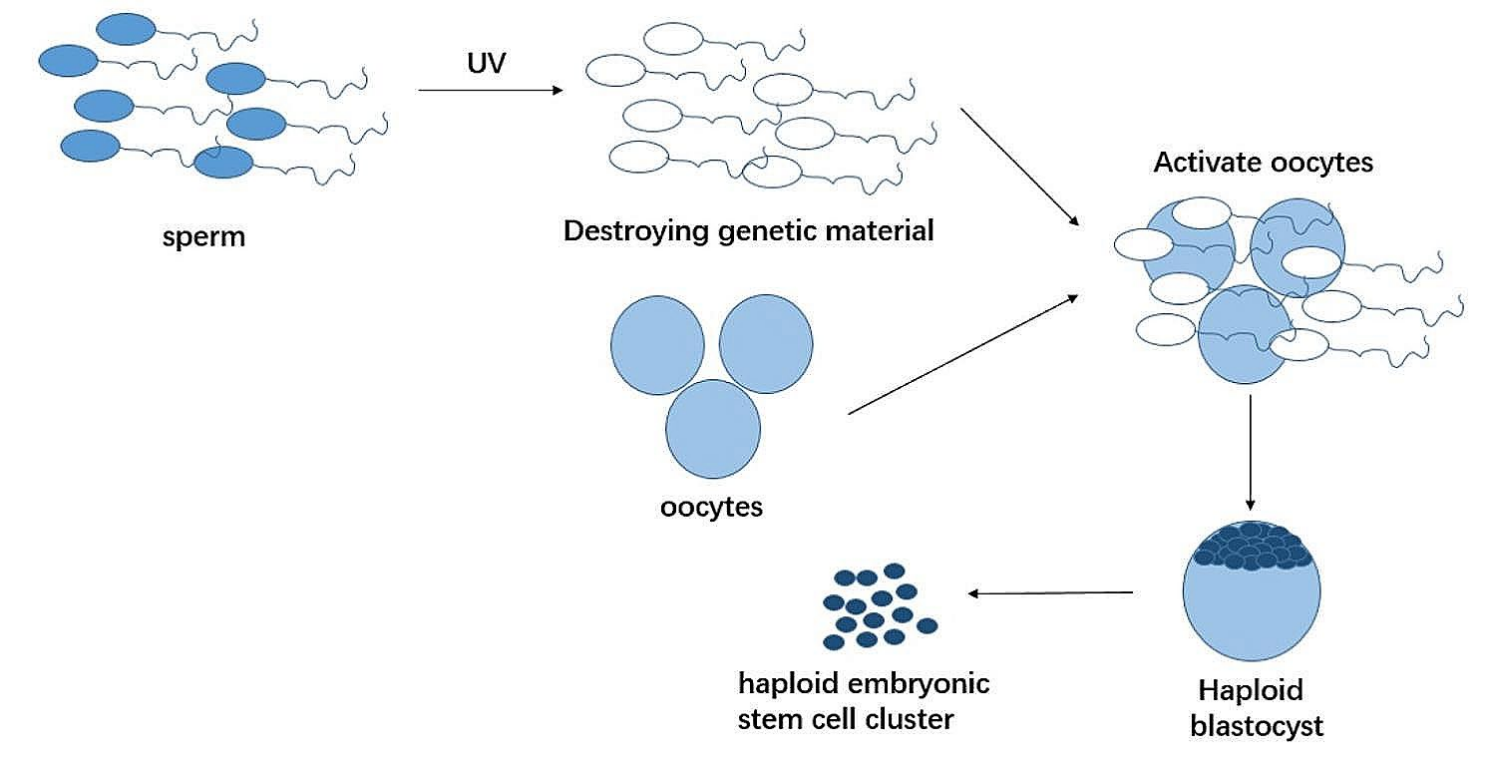
The process of obtaining medaka fish haploid embryonic stem cells

In recent decades, haESCs (haploid embryonic stem cells, haESCs) of different species have been established through a series of experiments (Table 1). In 2009, significant progress was made in obtaining haESCs in vitro [5]. In this study, using medaka fish as animal model, by inactivating the genetic material of the medaka fish sperm and mixing them with the oocytes, the oocytes were activated to start meiosis and finally developed into haploid embryonic stem cell cluster, thus establishing a relatively stable haploid embryonic stem cell line of vertebrates (Fig. 2). They then transplanted the genetic material of these haploid embryonic stem cells into the normal oocyte. In this way, the first semi-clone medaka fish was cultivated [5]. This method differs from conventional cloning in that half of the genetic material comes from haploid embryonic stem cells and the other half from normal oocytes. More excitingly, the first semi-cloned medaka fish can reproduce normally. This study demonstrates for the first time that vertebrate haploid cells can be cultured under specific conditions in vitro and have a growth and differentiation capacity similar to that of diploid cells, which was previously thought impossible.

In 2011, Elling et al. [6] and Leeb et al. [7] successfully established mouse parthenogenetic embryonic stem cells using different methods, which was the first time that a higher mammalian haploid embryonic stem cell line was established in vitro. Elling et al. [6] used chemical treatment of unfertilized oocytes to mimic the calcium oscillation caused by sperm entering the oocyte, making the parthenogenetic oocyte activation, and then transplanting the activated oocytes into pseudopregnant female mice. The cells of the cell mass in the blastocyst stage were amplified and further sorted and enriched by flow cytometry fluorescence sorting (FACS). Leeb et al. [7] obtained blastocysts in vitro. They activated unfertilized oocytes in vitro with Srcl2, cultured them in 2i medium with LIF (containing inhibitors of two apoptosis-related pathways -- kinase Mek pathway and kinase GSK3 pathway), and cultured the embryos to blastocyst stage, and further sorted and enriched haESCs in the same way. After subsequent verification, haESCs obtained by these two methods can maintain haploid stability. Their morphology, growth, and development ability are similar to normal diploid embryonic stem cells. They can express the classical embryonic pluripotency marker gene: klf4, 0ct4, Rex1, etc. They have the potential to differentiate into three germ layers and complete tissues. Despite the experiment’s limitations, a stable mouse parthenogenetic haploid embryonic stem cell line was successfully established, which laid the foundation for further research.

In 2012, the mouse androgenetic haploid embryonic stem cell line was established by Chinese scientists [8, 9]. In order to obtain the mouse androgenetic haploid embryonic stem cell line, the haploid androgenetic blastocysts need to be obtained first. For this, researchers used nuclear transfer technology: remove the nucleus of oocytes through microsurgery and inject single sperm into enucleated oocytes to form reconstructed haploid embryos that only carry the paternal genome. In further experiments, it was found that these embryos could develop into blastocysts in vitro, then isolated and established androgenetic haploid embryonic stem cell lines from these blastocysts. To verify whether the isolated male haploid embryonic stem cells obtained in the experiment can “fertilize” the oocytes like sperm, researchers injected the male haploid embryonic stem cell line into the oocytes. They found that part of the “fertilized” embryos developed into healthy mice. After that, haESCs of monkey and rat have also been successfully established, expanding haploid embryonic stem cell lines and further increasing the feasibility of mammalian haploid embryonic stem cells for gene research [10–12].

In 2016, the establishment of human haploid embryonic stem cell line also made a significant breakthrough, Sagi et al. [13] and Zhong et al. [14] used phase MII oocytes as experimental materials, activating phase MII oocytes by chemical activation and microsurgery, respectively, and established human parthenogenetic embryonic stem cell lines for the first time and verified the integrity and differentiation potential of its genome. In 2019, the more complicated human androgenetic haploid embryonic stem cells were successfully established by Chinese scientists [15]. They used micromanipulation technology to remove the nucleus of oocytes and successfully established human androgenetic haploid embryonic stem cells by sperm nuclear transfer. Recently, some experimental results have shown that male haploid embryonic stem cells with complete paternal imprinted genes in epigenetic inheritance have stronger development ability [16]. The establishment of haploid embryonic stem cell lines provides a new and powerful tool for developing human genetics, which is of great significance in human disease research, cell function, and other aspects. In addition, haESCs have strong self-renewal ability in vitro. They can be combined with advanced gene editing methods such as crisper-cas9 for gene editing to generate mammalian cells with homozygous alleles so that people can comprehensively study the function of recessive genes.

**Table 1:** Establishment of haploid embryonic stem cell line

origin	cell lines	founders
Mouse	Mouse Parthenogenetic haESCs (phESCs)	Elling U et al., 2011 [6]
		Leeb and Wutz., 2011 [7]
Mouse	Mouse Androgenetic haESCs (ahESCs)	Yang et al., 2012 [8]
Rat	Rat Androgenetic haESCs (ahESCs)	Li W et al., 2014 [11]
Rat	Rat Parthenogenetic haESCs (phESCs)	Li Z et al., 2016 [12]
Monkey	Monkey Parthenogenetic haESCs (phESCs)	Yang et al., 2013 [10]
		Wang H et al., 2018 [17]
Human	Human Parthenogenetic haESCs (phESCs)	Sagi et al., 2016 [13]
Human	Human Androgenetic haESCs (ahESCs)	Zhang, X.M., et al., 2019 [15]

## Haploid application

### haESCs and gene screening

**Fig. 3:**
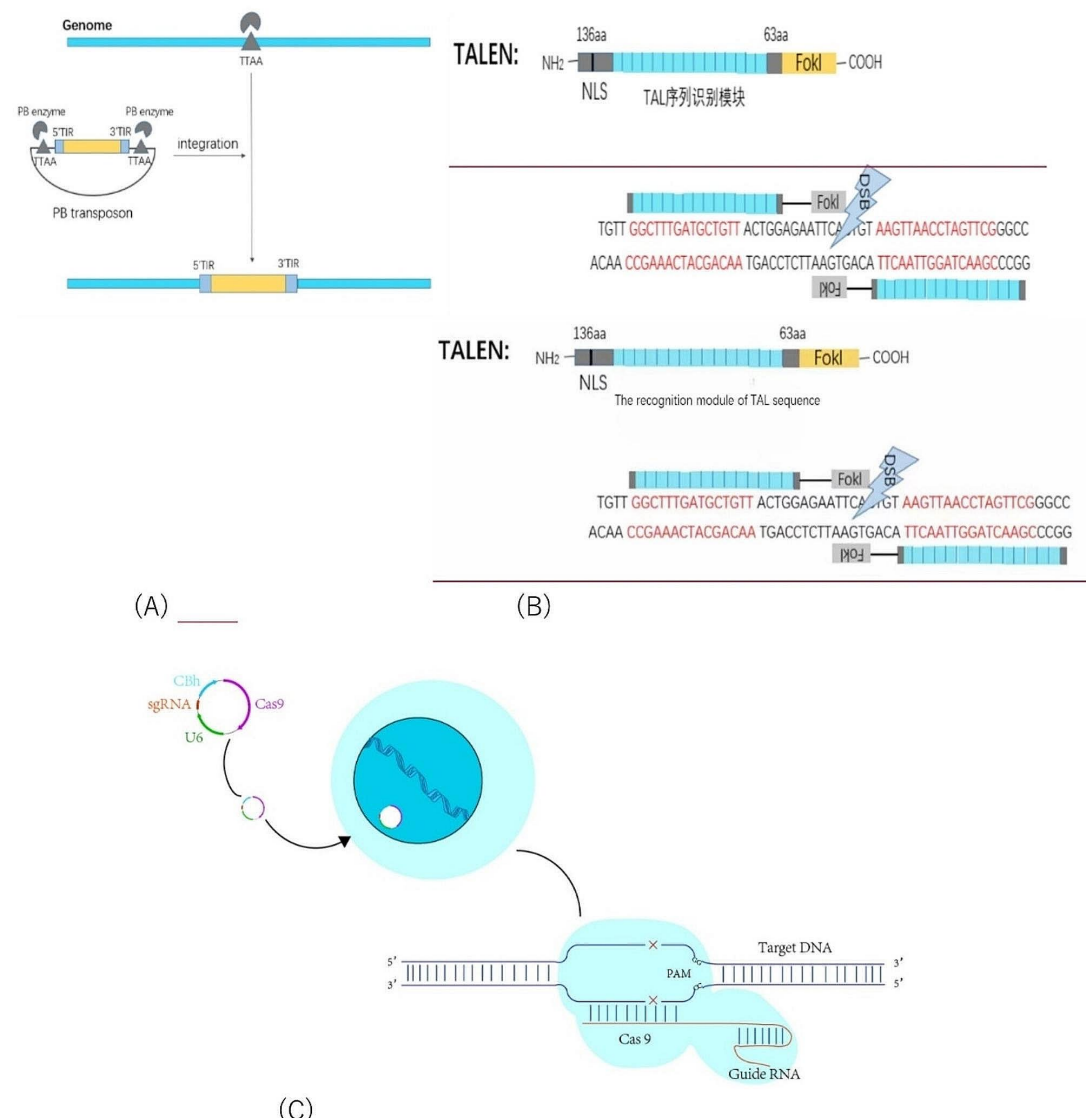
The mechanism of gene mutation: (**A**) induced by PB transposon (**B**) induced by TALENS (**C**) induced by CRISPR/Cas9

In the natural state, it is challenging to obtain homozygous mutations in diploid embryonic stem cells, and it is difficult to show heterozygous recessive genes in alleles, so the diploid genome in the natural state hinders the research on the function of recessive genes. However, it is time-consuming and laborious to obtain homozygous allele mutations using gene editing technology, so studying the function of recessive genes is difficult. The use of haESCs can overcome this obstacle, for haploid cells only carry one set of chromosomes, they can show corresponding phenotypes after mutation, making haESCs widely used in functional genomics research and genetic screening. Generally, researchers use the yeast of nature haploid cells to screen mutations within the whole genome [18–20]. However, this method cannot be applied to mammals due to species specificity. With the greatly reduced difficulty of obtaining mammal haESCs, genome-wide screening has been widely used in mammals to clarify the functions of various genes in different biological processes, which has extensively promoted the development of medical and pharmacological research. Traditional genetic screening has two ways: forward genetics and reverse genetics. In general, forward genetic screening is to obtain the phenotype of loss of function through allele mutation and then study the function of alleles, that is, from phenotype to gene. Reverse genetic screening tracks phenotypic changes through changes in a specific gene or protein, that is, from gene to phenotype. HaESCs can be widely used in forward genetic screening through genetic engineering. Genome engineering can generate mutant libraries through transposon-mediated insertion or nucleic acid-mediated targeted modification technologies, including piggyBac transposon, CRISPR/Cas9 gene editing technology, and TALENS (Fig. 3) etc. After gene editing, haESCs can obtain specific mutant cells through screening and verification.

So far, researchers have made some progress in gene function and clinical disease research through gene screening of haploid cell lines [21]. For example, in 2022, researchers used haploid embryonic stem cells for gene screening and screened out essential genes related to pluripotency [22]. They used the haploid system to screen the whole genome and found that Thop1 is the key factor for the pluripotent withdrawal of rat embryonic stem cells. They used piggyBac transposon to make the mutation cover the whole genome of Rex1-GFP haploid embryonic stem cells in rats. They then performed a random differentiation experiment to screen the differentiation delayed mutation library. Combined with high-throughput sequencing and bioinformatics analysis, the mutation sites were clarified one by one. Finally, the role of candidate mutant genes in rat embryonic stem cells was demonstrated by knockout and over-expression experiments. This experiment shows that a homozygous haploid system carrying a pluripotency reporter gene is an ideal tool for studying rat functional genomics, which helps discover the relevant mechanisms of self-renewal or pluripotency of rat embryonic stem cells. Besides, the research team used haploid embryonic stem cells to conduct genome-wide screening and found the target gene of oxidative toxicity - Slc25a43 [23]. They also used haploid epiblast stem cells, revealing Hs3st3b1 as a modulator for reprogramming [24]. Some laboratories combined CRISPR/Cas9 gene editing technology with haploid embryonic stem cells to explain the function of genes. For example, Shiran Bar et al. applied CRISPR/Cas9 gene editing technology to haploid embryonic stem cells to screen the regulatory factors of parental imprint [25] (Table 2).

**Table 2:** Haploid cells for genetic screening

Cell type	Mutant method	Target gene	Target gene function	reference
Mouse haESCs	retrovirus	Gpr 107	Ricin toxicity	Elling, U., et al. [6]
Mouse haESCs	retrovirus	SPEN	X-chromosome inactivation	Monfort, A., et al. [26]
Human haESCs	retrovirus	NUDT5	Resistance to 6-TG	Sagi, I., et al. [13]
Mouse haESCs	PB	Msh2, Hprt	Resistance to 6-TG	Leeb, M. and A. Wutz [7]
Mouse haESCs	PB	Parp1	Resistance to olaparib	Pettitt, S.J., et al. [27]
Monkey haESCs	PB	PRKD1 et al.	Detection of mutation efficiency	Yang, H., et al. [10]
Mouse haESCs	PB	2fp706, Pum1	Exit from self-renewal	Leeb, M., et al. [28]
Mouse haNSCLSs	PB	Park2	Mn^2+^ toxicity	He, Z.Q., et al. [29]
Monkey haNPCs	PB	B4GALT6	Tetrodotoxin-like toxicant	Wang, H., et al. [30]
Mouse haiTSCs	PB	Htra1	Blocker for spongiotrophoblast specification	Peng, K., et al. [31]
Mouse haTSCs	PB	Hprt	Resistance to 6-TG	Cui, T., et al. [32]
Rat haESCs	PB	Thop1	Related to pluripotency	Xu, M., et al. [22]
Mouse haESCs	PB	Slc25a43	Oxidative toxicity	Zhang, J., et al. [23]
Mouse haEpiSCs	PB	Hs3St3b1	Related to reprogramming	Gao, Q., et al. [24]
Mouse haESCs	EMS	Hprt	Resistance to 6-TG	Forment, J.V., et al. [33]
Mouse haESCs	CRISPR/Cas 9	Zicl, Clec11a, et al.	Related to bone development	Bai, M., et al. [34]
Mouse haESCs	CRISPR/Cas 9	4 amino acids of DND1	Related to stability of DND1 protein	Li, Q., et al. [35]
Human haESCs	CRISPR/Cas 9	ATF7IP	Related to maternally imprinted	Bar, S., et al. [25]
Human haESCs	CRISPR/Cas 9	p53	resistance to a large array of anticancer drugs	Segal, E., et al. [36]
Human haESCs	CRISPR/Cas 9	BRCA2	sensibility to PARP inhibitors	Li, H., et al. [37]
Human haNSCs	PB	SHANK2, KMT5B, et al.	manganese-induced toxicity genes	Wang, HS., et al. [21]
Mouse haESC	PB	Catip, Dyrk1a	Conversion from ESC to TSC	Zhang, W., et al. [38]

### haESCs and X chromosome inactivation

HaESCs have the potential application as a tool cell for studying the mechanisms related to X chromosome inactivation (XCI). There is a gene dose compensation effect in the mammalian XY sex determination mechanism. Because of this effect, sex-link genes have equal or nearly equal effective doses in different genders, and the epigenetic modification of XCI is closely related to the gene dose compensation effect [39]. Under the XCI mechanism, one X chromosome in diploid female somatic cells is in the transcriptional activation state (Xa), and the other X chromosome is in the silent state (Xi) after epigenetic modification. The corresponding female diploid embryonic stem cells can show two XCI states, XaXa, and common XaXi [40]. The mechanism of XCI is closely related to the expression of long-chain non-coding RNA Xist, which can inactivate one X chromosome at random [41, 42]. Monfort et al. [26]screened the RNA binding protein SPEN, a key regulatory factor in the XCI process, by inducing Xist overexpression. This experiment shows that the RNA binding protein SPEN is a key factor for Xist playing a role in gene inhibition. In the process of XCI mechanism research, haESCs can be used as a tool cell for genetic screening to identify the role and pathway of SPEN in Xist gene silencing.

Recently, a research team used mouse haploid embryonic stem cells to study the activation process of Xist [43]. Li et al. [44] successfully constructed mouse rat allogeneic hybrid diploid embryonic stem cells (AdESCs) by fusion of mouse and rat haESCs. The heterodiploid genome of these AdESCs is relatively stable and has the universal characteristics of diploid cells. Surprisingly, they found that almost all X-linked gene transcripts sequenced existed on the X chromosome of rats, while the transcripts of the silent Xist gene only existed on the X chromosome of mice. These results indicate that the mouse X chromosome is inactivated in heterodiploid cells in mouse and rat fusion. They also systematically analyzed a large number of RNA-seq data and found 146 unknown mouse genes in heterodiploid somatic cells, which may be related to X chromosome inactivation escape. The other research team [43] obtained haploid primordial germ cell-like cells (PGCLCs) from haploid mouse embryonic stem cells (ESCs). They find that haploid cells show predisposition for PGCLCs, whereas a large fraction of somatic cells becomes diploid. Characterization of the differentiating haploid ESCs (haESCs) reveals that Xist is activated from and colocalizes with the single X chromosome. This observation suggests that X chromosome inactivation (XCI) is initiated in haploid cells consistent with a model where autosomal blocking factors set a threshold for X-linked activators. They further find that Xist expression is lost at later timepoints in differentiation, which likely reflects the loss of X-linked activators. This research suggests that in vitro differentiation of haploid PGCLCs can be a useful approach for future studies of potential X-linked activators of Xist. In 2023, researchers have proven that “haESCs had transcriptome profiles closer to those of naïve pluripotent stem cells. Consistent with the one X chromosome in haESCs, Xist was repressed, indicating no X chromosome inactivation.” [45].

The above research results confirm that haESCs are a powerful tool for studying the relevant mechanisms of X-chromosomal inactivation and screening XCI inactivation escape genes.

### haESCs and unisexual reproduction

**Fig. 4:**
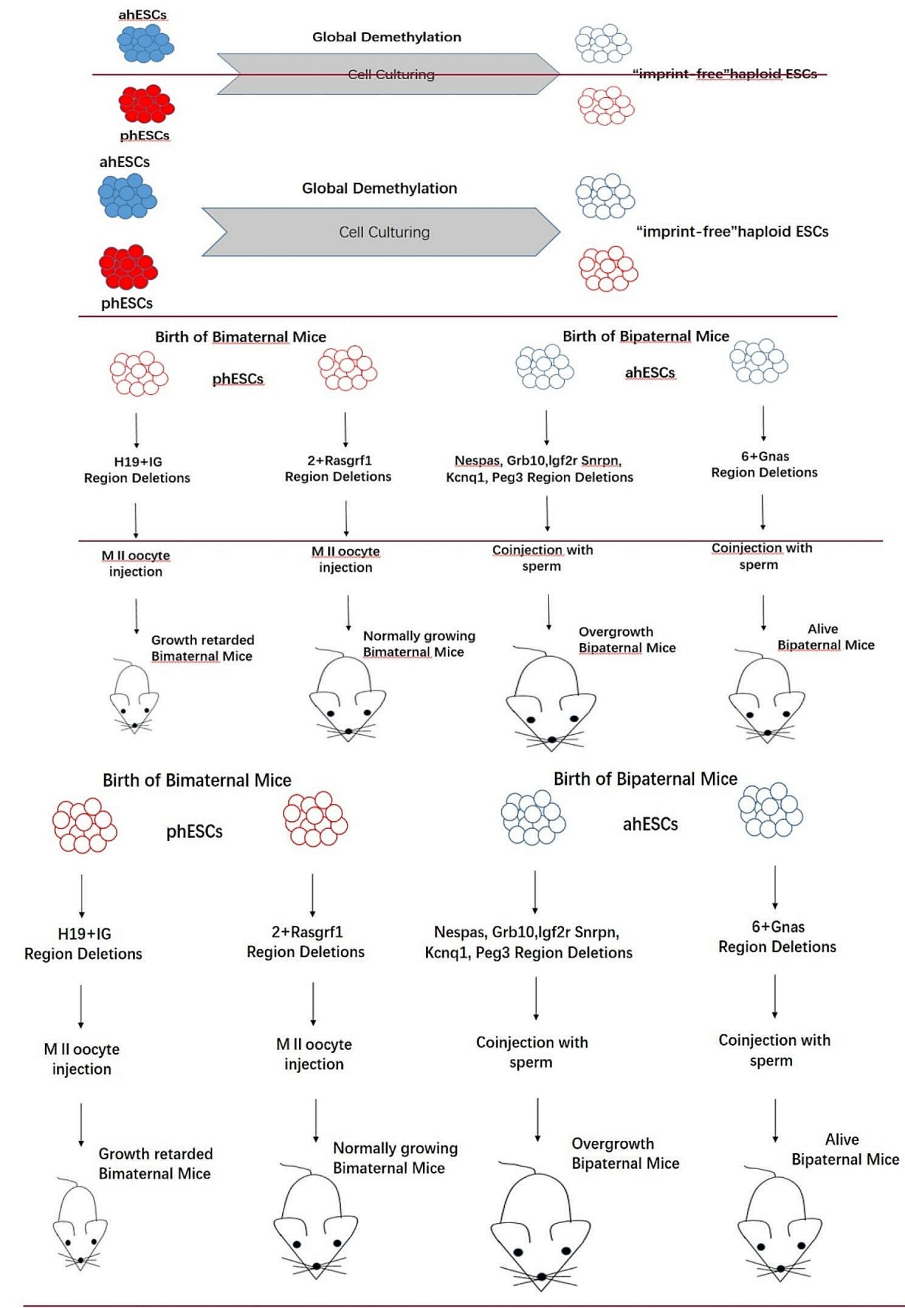
Generation of Bimaternal and Bipaternal Mice from Hypomethylated Haploid ESCs with Imprinting Region Deletions

Unisexual reproduction includes bimaternal reproduction and bipaternal reproduction. Bimaternal reproduction means an egg develops into a new individual through DNA replication without fertilization. Compared with bipaternal reproduction, bimaternal reproduction is more common in nature, such as in lizards, frogs, and fish [46–48]. In contrast, bipaternal reproduction is only found in very few lower animals [49]. At present, unisexual reproduction has not been found in higher mammals. This is because the imprinted genes of parents hinder the normal development of unisexual embryos [50]. Therefore, the realization of bimaternal reproduction must overcome the impediment of imprinted genes. In 2004, Japanese scientist Kono et al. [51] obtained the surviving bimaternal mice by using immature oocytes lacking the H19 imprinted gene for the first time, they achieved parthenogenesis in higher mammals for the first time. This experiment result also confirmed that imprinted genes obstruct parthenogenesis in mice. In 2007, Kawahara et al. [52] improved the birth rate of bimaternal mice by deleting H19-DMR and IG-DMR. Because eggs and sperm are highly specialized cells in structures and functions, with the establishment of haploid embryonic stem cell lines of various organisms, researchers realized the benefits of haESCs as gamete substitutes to generate unisexual animals. Although bimaternal mice have been successfully obtained, it is still extremely difficult to obtain bipaternal mice. Until 2018, Li et al. [53] took the lead in making progress in the research of acquiring bipaternal mice. In order to get living bipaternal mice, they knocked out seven imprinted regions including the Gnas immunoblot region. They acquired 12 bipaternal mice from 477 embryos using tetraploid compensation, which is the first time in the world to obtain bipaternal mice with two paternal genomes, this research results confirmed the feasibility of unisexual reproduction in higher mammals. Although 10 of the 12 bipaternal mice survived less than 2 days, the remaining 2 failed to survive to adulthood (Fig. 4.). The team used haESCs as a research tool to explain the necessary factors for mammals to overcome unisexual reproduction disorders. This experiment shows that constructing an embryo system with haESCs as gamete substitution can be used as a “springboard” to study the regulation mechanism of embryo development, broadening the depth and breadth of the application of haESCs. Recently, in order to obtain de novo male gametes capable of inducing full preimplantation blastocyst development, research teams using a novel three-dimensional (3D) culture system for culturing Mouse embryonic stem cells (mESCs) during the generation of functional gametes [54–56].

## Maintenance and screening of Haploid

Haploidy is the unique advantage of haploid cells in gene application. Still, the spontaneous diploid characteristics of haploid cells limit their application in gene research, so it is essential to maintain haploidy in haploid cell application [57]. The maintenance of haploidy can be achieved through FACS enrichment and sorting [58]: First, using Hoechst 33342 to dye embryonic stem cells. Under laser irradiation with a wavelength of 355 nm, the nucleus emits fluorescence with a wavelength of 461 nm. The content of haploid DNA is only half that of diploid cells, so the fluorescence signal is also significantly weaker than that of diploid cells. Then flow cytometry was used to enrich haploid cells according to nucleic acid content. According to this feature, the cells can be divided into three peaks in the flow cytometry, namely 1 C (G0/G1 haploid cells), 2 C (G2/M haploid cells and G0/G1 diploid cells), and 4 C (G2/M diploid cells). The 1 C peak is the haploid cells, which can be separated from the haploid cells more accurately by this method [59]. However, FACS is a high-cost and complex technology that depends on large equipment. Besides, the cytotoxicity of Hoechst 33342 dye and ultraviolet radiation will reduce the survival rate and affect the acquisition efficiency [60]; these factors limit the broad application of FACS. Recently, it has been reported that there are noticeable size differences between cells with different DNA contents. According to this feature, researchers have developed a method to enrich haploid cells by simple filtration. They separate haploid cells with smaller diameters from diploid cells by regular filtration. This method is relatively fast and straightforward, with less physical damage to cells, and is more economical and applicable, making maintenance of haploid embryonic stem cell lines easier [61, 62]. Using FACS technology to sort and enrich haploid embryonic stem cells has high accuracy, but its complexity and high-cost limit its use. The method of enrichment of haploid embryonic stem cells by simple filtration has the advantages of low coefficient of difficulty, fast and simple, economical and applicable. Still, its accuracy needs to be further improved. We look forward to furthering technical improvement and reform in the future, which is of great significance for applying haploid embryonic stem cells.

## Haploid diploidization

It is precise because haploid embryonic stem cells have the characteristics of homozygous phenotype and pluripotency of stem cells that they can be rapidly and accurately applied to explore the unknown functions of novel genes. However, haploid embryonic stem cells have the characteristic of spontaneous diploid in the culture or differentiation process, thus losing the advantage of a single copy genome [63]. However, haploid cells need to be sorted and enriched periodically by time-consuming and complex sorting methods, which greatly limits the application of haploid stem cells in many fields. Therefore, exploring the diploidization mechanism of haploid stem cells and inhibiting this pathway can fundamentally maintain the haploid of haploid stem cells, which is of great significance for promoting haploid embryonic stem cells as tool cells.

At present, some research results show that the abnormal cell cycle, rather than the fusion between haploid cells, is the mechanism of spontaneous diploid. The mitotic process of normal diploid cells can be divided into interphase and mitotic phase (M phase). The interphase is also divided into the G1 phase (synthesis of RNA and protein), the S phase (DNA synthesis in the nucleus doubles DNA) and the G2 phase (cells further grow and synthesize protein). However, some haploid cells are abnormal during the mitotic phase, skipping the M phase and entering the G1/S phase again, so the DNA content in the cells becomes twice the original [64, 65]. Takahashi et al. [64] found that adding Wee1 kinase inhibitor to the culture medium can accelerate the transition from the G2 phase to the M phase of haESCs and prevent the entry of additional G1/S phase. This method can effectively increase the maintenance time of haESCs in vitro. Other researchers found that PD 166285 (Wee1 kinase inhibitor) and RDF (R: Repsox, TGF-β Pathway inhibitors; D: DMH1, BMP4 pathway inhibitor; F: Forskolin, an adenosine cyclase activator) were added to 2i medium, can accelerate the transformation of haESCs from S/G2 phase to M phase, thereby increasing its haploidy maintenance time to more than 5 weeks. Recently, experimental results have shown that the prolongation of the replication time of haploid embryonic stem cells leads to the change of dynamics in the S-phase, which affects the whole cell cycle and leads to the phenomenon of haploid diploidization. The prolongation of replication time is related to the lack of X chromosome inactivation of haploid embryonic stem cells [66].

In 2020, researchers [67] further discovered the diploid mechanism of haploid embryonic stem cells. This study found that inhibition of apoptosis can reduce the doubling phenomenon of mouse haploid embryonic stem cells in daily culture and differentiation in vitro and in vivo. This study induced p53- knockout haESCs to enter multiple differentiation lineages and maintain haploid status in vitro. Transcriptome analysis showed that apoptotic genes in p53- knockout haESC were downregulated compared with wild-type haESCs. In another experiment, they knocked out another apoptosis-related gene, p73 and observed the stable phenomenon of haploid in haESCs. These results indicate that the primary mechanism of diploidization is cell death triggered by apoptosis-related genes in haploid cell culture. Therefore, haploid cells can be obtained by manipulating apoptosis genes to promote genetic screening following specific development. In this study, the p53-knockout was used to stabilize the haploidy of haploid embryonic stem cells (haESCs). It was found that p53-knockout haESCs (p53-KO haESCs) could obtain highly haploid embryoid, epigenetic stem cell like cells, and neural stem cell like cells after differentiation in vitro. The differentiation experiment in vivo proved that p53-KO haESCs could contribute to the 6.5, 8.5, and 10.5 days of embryonic development through the chimerism experiment and maintained a high haploid proportion. In addition, haploid somatic cells can also be detected in teratoma differentiation experiments in vivo. In order to explore the mechanism of p53-KO haESCs’ stable maintenance of haploidy, through transcriptome sequencing analysis, the author found that compared with wild-type haESCs, p53-KO haESCs had significantly decreased the expression of some apoptotic genes, while no significant difference was found in the gene expression levels of other signal pathways related to p53. The subsequent results showed that the deletion of apoptosis gene p73 could also effectively maintain the haploidy of haESCs. This study proves that p53 knockout mainly maintains the haploidy of haESCs in the process of daily passage culture and in vitro and in vivo differentiation by inhibiting apoptosis, provides a fast and effective strategy for obtaining various haploid differentiated cells, and promotes the genetic screening research of haploid cells in various lineages. Recently, studies have shown that haploid embryonic stem cells with Etl4 gene knockout (Etl4-KO) are more likely to maintain haploidy [68]. Recently, research shows “overexpression (OE) of an antiapoptosis gene, BCL2, in haESCs robustly ensures their haploidy maintenance in various situations, even under strict differentiation in vivo (embryonic 10.5 chimeric fetus or 21-day teratoma).” [69].

## Conclusion

The successful establishment of haploid embryonic stem cell lines from fish, mice, rhesus monkeys, and rats to humans has expanded haploid embryonic stem cell lines step by step, thereby expanding the research scope of haploid embryonic stem cells. With the continuous deepening of research on human disease mechanism, cell function, and reproductive development, haESCs, as a powerful tool, although there are still many problems to be solved in the establishment, maintenance, and application of cell lines, will continue to develop with its research content deeply. In a word, the research on haESCs has made some progress, but there is still much work to be done, which will greatly promote the application of haploid cells in genetic analysis.

## Data Availability

Not applicable.
